# The Efficacy of Lumbar Support on Pain, Disability, and Motor Control in Women With Postpartum Pelvic Girdle Pain: Protocol for a Randomized Controlled Trial

**DOI:** 10.2196/40553

**Published:** 2022-07-20

**Authors:** Fahimeh-Sadat Jafarian, Mahmonir Jafari-Harandi, Gillian Yeowell, Ebrahim Sadeghi-Demneh

**Affiliations:** 1 Musculoskeletal Research Center School of Rehabilitation Sciences Isfahan University of Medical Sciences Isfahan Iran; 2 Department of Obstetrics & Gynecology School of Medicine Isfahan University of Medical Sciences Isfahan Iran; 3 Department of Health Professions Manchester Metropolitan University Manchester United Kingdom

**Keywords:** sacroiliac joint, pain, disability, motor control, lumbosacral orthosis, postpartum

## Abstract

**Background:**

Pregnancy-related posterior pelvic girdle pain (PPGP) is one of the most important clinical manifestations of postpartum back pain. Those affected often complain of discomfort during daily activities. It is hypothesized that altered motor control is associated with perceived pain. Pelvic support can regulate possible underlying altered motor control mechanisms and decrease pain. However, the influence of a lumbosacral orthosis, which is broader support that allows for a wider contact area and more skin sensory stimulation to restore proper motor function, has not yet been investigated in women with postpartum PPGP.

**Objective:**

This study investigates the efficacy of broader lumbar support and narrower pelvic support on pain, proprioception, disability, and muscle strength in women with pregnancy-related PPGP.

**Methods:**

This study will be a single-center, 3-armed, participant-blinded, randomized controlled trial. In total, 84 women diagnosed with pregnancy-related PPGP will be recruited and randomly assigned into 3 groups. Intervention groups A and B will receive pelvic and lumbar supports, respectively. Group C (control) will receive only a patient education leaflet containing advice on strengthening exercises, comfortable positions, and other practical information. The study outcomes are pain, effort score during the active straight leg raising test, maximum isometric hip flexion force, maximum isometric hip external rotation force, maximum isometric trunk rotation force, and joint position reproduction of hip abduction. The study outcomes will be measured at 4 time points: baseline (T1), immediately after the intervention (T2), 4 weeks following interventions began (at this time, the intervention period is completed) (T3), and 1 week after discontinuing the interventions (T4) to evaluate the possible lasting effects of wearing supports. Multivariate analysis of variance will be used to test between- and within-group differences.

**Results:**

Recruitment for this study will be started in summer 2022 and is expected to be completed by the end of fall 2022.

**Conclusions:**

This study will examine the efficacy of broader lumbar support as an early rehabilitative treatment for women receiving postpartum posterior pelvic pain support compared to those receiving a narrower pelvic support. We expect the broader lumbar support to impact pain management and disability better than the current narrower pelvic belt. Long-term follow-up studies will help determine whether such lumbosacral orthosis reduces pain and improves daily activities in women with pregnancy-related PPGP.

**Trial Registration:**

Iranian Registry of Clinical Trials IRCT20150210021034N11; https://www.irct.ir/trial/54808

**International Registered Report Identifier (IRRID):**

PRR1-10.2196/40553

## Introduction

Pregnancy-related posterior pelvic girdle pain (PPGP) is a common musculoskeletal disorder affecting women’s well-being in the postpartum period [1]**.** Nearly half of the involved population demonstrates moderate to severe disability [2]. This painful condition is related to physical and psychosocial aspects, including kinesiophobia, psychological distress, beliefs regarding curability, financial stress, and social isolation [3-6]. The alteration of motor control in people with pelvic pain has been documented [7] and can affect a change in the load transfer ability at the pelvis [8]. Pregnancy-related PPGP is commonly localized in the vicinity of the sacroiliac joint (SIJ) [9]. One of the SIJ’s functions is transmitting the upper body weight to the pelvis and lower extremities and vice versa [10]. Dynamic control of mechanical and neural systems acting on the pelvis allows the lumbopelvic movements to be smooth, painless, and effortless under changing conditions [11]. Pregnancy-related PPGP includes the dysfunction of sensorimotor pathways that control load transfer through the SIJ [11,12]. The altered spinal and abdominal muscle activations and perceived pain affect the function of the SIJ in postnatal women [13]. Those with pregnancy-related PPGP often complain of discomfort during load transfer tasks through the pelvis [14]. The active straight leg raise (ASLR), as a biomechanical test, is proposed to check load transfer between the spine and legs via the pelvis [14,15].

Many preventive or therapeutic strategies are used for the management of pregnancy-related PPGP. Current pain relief recommendations include avoiding certain physical loads, relaxation, massage, medications, exercise, physical therapy, and pelvic or lumbar supports [16]. Among the recommendations for managing pregnancy-related PPGP, it was noted that pelvic or lumbar support is widely used to manage painful symptoms [17,18]. Pelvic or lumbar support, also known as soft orthosis, can work through a combination of mechanisms to address the SIJ instability. First, the circumferential supports are expected to apply a compressive force on the pelvis to promote anatomical alignment [19], stiffness [20], and increase the function of the SIJ [21]. Second, soft orthoses can also provide potent stimuli to improve neuromuscular control required for lumbopelvic function [22]. It has been shown that the gentle pressure soft orthoses applied to the skin receptors can positively affect proprioceptive acuity [20,23], pain intensity [17,24], joint stability [25,26], and physical function [17,27].

Emerging research has highlighted the sensorimotor effectiveness of narrow pelvic supports in pregnancy-related PPGP [27,28]. Furthermore, previous research has indicated that lumbar support effectively improved proprioceptive awareness in healthy subjects [22]. However, the effect of broader lumbar supports that allow for a wider contact area and greater skin sensory stimulation in women with pregnancy-related PPGP remains unclear. Therefore, this study first aims to investigate whether using lumbar support reduces pain in women with pregnancy-related PPGP versus controls. Secondly, it aims to examine whether broader lumbar support is more beneficial than narrower pelvic support on disability and sensorimotor outcomes (eg, joint positions reproduction) in the lumbopelvic area in women with pregnancy-related PPGP.

## Methods

### Study Design

A prospective 3-armed, participant-blinded, randomized controlled trial (interventions versus control group) will be performed to investigate the aims. The Template for Intervention Description and Replication (TIDieR) checklist and related guidelines will be used to report the study process [29,30]. The TIDieR checklist describes a standard way to document details carefully and allows authors to write interventions in their studies clearly [29]. The CONSORT-EHEALTH (Consolidated Standards of Reporting Trials of Electronic and Mobile Health Applications and onLine TeleHealth) checklist will be used to report data efficiently as an eHealth intervention trial [31].

### Setting

Participants will be recruited from Isfahan University of Medical Sciences (IUMS), Isfahan, Iran.

### Ethics Approval

The local ethical committee of IUMS approved the study protocol (IR.MUI.NUREMA.REC.1400.007). The study protocol was registered in the Iranian Registry of Clinical Trials on April 31, 2021 (IRCT20150210021034N11).

### Sample Size

In total, 84 women with postpartum PPGP will be recruited. The optimal sample size was calculated on the basis of the results of a previous study [32]. The pain intensity for intervention groups was 58.2 (SD 13.93) and 64.4 (SD 13.96) at baseline, respectively [32]. We estimated a sample size of 25 in each study arm, which would yield 60% power (α=.1) and an effect size of 0.44 (Cohen *d*) in accordance with calculations performed using G*power software (Version 3.1, Universität Düsseldorf). We also considered an overall dropout rate of 10% (eg, lost to follow-up). Therefore, we aimed to recruit 84 participants (28 in each study arm).

### Participants

Women with postpartum PPGP will be recruited through a simple (convenience) sampling method from the obstetric outpatient clinics of IUMS. The examiner will confirm the diagnosis with the presence of PPGP, and positive diagnostic tests include the following: ASLR, posterior pelvic pain provocation (also known as P4) test, Patric-Faber test, and Gaenslen’s test [33]. The inclusion criteria of the study are (1) primipara women who experienced natural delivery (one month before); (2) age between 18 and 45 years; (3) self-reported pregnancy-related PPGP; (4) a pain score of at least 40 out of 100 mm on the visual analog scale (VAS) [17]; and (5) a score of higher than 2 out of 5 on a 6-point Likert scale for perceived effort during the ASLR test [34]. The exclusion criteria were as follows: (1) the presence of lower back or pelvic pain before pregnancy; (2) history of any fracture in the pelvis and lower extremities; (3) history of spine, pelvis, and lower extremity surgery; (4) neurological diseases; (5) limb length discrepancy; (6) congenital anomaly in the spine, pelvis, and lower extremities; and (7) using any other conservative treatment for pain relief during the study, such as physiotherapy treatment methods. All eligible participants will provide informed consent and sign the consent form before inclusion in the trial. Participants will be free to withdraw at any time during the study.

### Assignment, Randomization, and Blinding (Masking) Procedures

Once participants are confirmed to be eligible, they will be randomly assigned with equal allocation at a 1:1:1 ratio (one control participant per treatment participant) to one of 3 groups: (A) pelvic (narrower) support (n=28), (B) lumbar (broader) support (n=28), and (C) control group (patient-education leaflet) (n=28). Block randomization will be used, with a block size of 6 to achieve balance in allocating participants to study arms [35]. The assessor will perform randomization with a Random Allocation Software (version 1.0) [36]. The nature of this study will not allow for masking of the assessor after assignment to interventions. Only participants will be masked to group assignment at the point of allocation.

### Adverse Events and Dropouts

Adverse events will be mentioned and recorded throughout the trial for participant safety. Information about adverse events, including the date and participant’s experience, will be summarized in tables. Information about participants who discontinued the study will be recorded. Furthermore, the reasons for dropout, such as COVID-19 symptoms, loss to follow-up, and sickness, will be reported.

### Study Arms and Content

**Figure 1:**
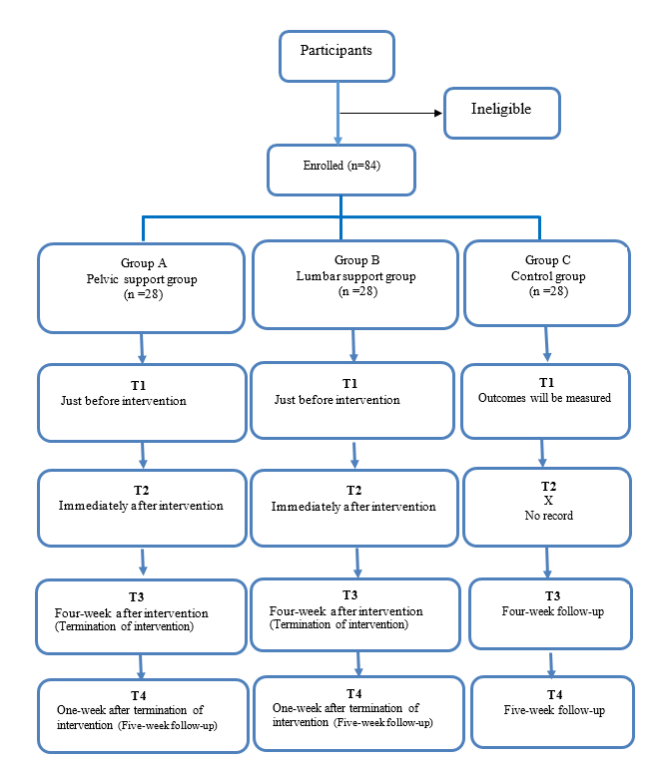
The study flowchart for the randomized controlled trial.

There will be 3 different study arms. As intervention groups (A and B), the first and second arms will receive pelvic or lumbar supports for 4 weeks. As the control group (C), the third arm will only receive a patient education leaflet containing advice on strengthening exercises, comfortable positions, and other practical information. The outcomes include pain, effort score during the ASLR test, maximum isometric hip flexion force, maximum isometric hip external rotation force, maximum isometric trunk rotation force, and joint position reproduction (JPR) of hip abduction. The study outcomes will be measured at 4 time points: baseline (T1), immediately after the intervention (T2), 4 weeks after the interventions began (at this time, the intervention period is completed) (T3), and 1 week after discontinuing the interventions (T4) to evaluate the possible lasting effects of wearing supports. The study process is outlined in Figure 1.

### Intervention

**Figure 2:**
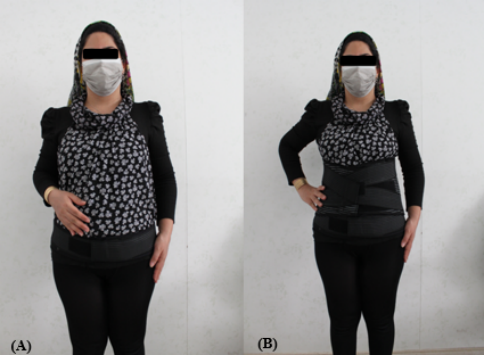
Study interventions: (A) pelvic support and (B) lumbar support.

This study will use 2 orthoses: pelvic (Group A) and lumbar (Group B) support (Teb Darman Co). Both orthoses are made of breathable textile material to comfort the participant. Orthoses can be worn on top of or under daytime clothes. The pelvic support will be an adjustable strap (10-15–cm width) fastened below the anterior superior iliac spine (Figure 2A). The lumbar support consists of a pelvic belt attached to the lumbar corset. Lumbar support has a 25-cm width anteriorly and extends from the xiphoid process to the pelvis. It has a 35-cm width posteriorly and extends down from the lower angle of the scapula to gluteal prominences (Figure 2B). Group C (control) will receive only a patient education leaflet containing advice on strengthening exercises, comfortable positions, and other practical information. Participants will be instructed to incorporate the regular activities into their daily routines and not use pain relief medication or other treatment during the following 5 weeks. Although we do not expect participants to experience any trial-related harm, they will be provided with a direct number to contact should they have any questions or queries and report any symptoms related to interventions or any discomfort experienced while wearing their orthoses.

### Outcome Measures

All tests will be carried out with a certified orthotic practitioner. Table 1 outlines the outcome measures and shows how outcome measures will be measured at different time points throughout the study.

**Table 1:** The outcome measures of the study.

Measures	Baseline (T1)	Immediate effect (T2)	Fourth week (T3)	Fifth week (T4)	
Overall pain	✓	✓	✓	✓	Visual analog scale
Effort score during active straight leg raise	✓	✓	✓	✓	6-point Likert scale
Maximum isometric hip flexion force	✓	✓	✓	✓	Digital force gauge
Maximum isometric trunk rotation force	✓	✓	✓	✓	Digital force gauge
Maximum isometric hip external rotation force	✓	✓	✓	✓	Digital force gauge
Joint position reproduction of hip abduction	✓	✓	✓	✓	Kinovea software
Activity limitation	✓	✗	✓	✓	Modified Oswestry Disability Index

### Primary Outcome Measures

#### Pain

The participant will indicate the severity of pain related to the lumbopelvic region during the previous week on a 10-point VAS ranging from 0 to 10, where 0=no pain and 10=the worst imaginable pain [24]. The VAS is documented and recommended as reliable in subjects with lower back pain (SE 0.09, intraclass correlation coefficient [ICC] 0.90) [37].

#### Modified Oswestry Disability Index for Lower Back Pain

The Persian version of the Oswestry Disability Index (ODI) will quantify disability in women with postpartum PPGP [38]. This self-reported 10-item questionnaire has been introduced as a valid and reliable tool compatible with spine-related disabilities [39]. The first item evaluates the pain intensity. The other items ask about pain intensity experienced while carrying out typical daily activities, including personal care, lifting, walking, sitting, standing, sleeping, social life, traveling, and employment/homemaking. Each item scored 0-5; higher scores imply higher pain intensity and disability. The ODI has been introduced as a gold standard for lower back functional outcome tools and has good psychometric properties, which can be used in various settings [40]. The ODI shows a good construct validity, acceptable internal consistency (Cronbach α=.69-.87) [38,41,42], and high test-retest reliability (*r*=0.83-0.99) in people with lower back pain [41-43].

### Secondary Outcome Measures

#### Effort Score During ASLR

With the participant lying in supine position on the examination table, they will be instructed to keep their knee straight with their feet 20 cm apart during tests. The raising height during the ASLR test will be defined by placing a metal bar 20 cm above the examination table (target position) [44]. The perceived effort will be scored while the participant raises their leg to the target position. They will indicate their perceived difficulty in performing the test on a 6-point Likert scale ranging from 0 to 5, where 0=no problem and 5=unable to do [44]. The test-retest reliability of the ASLR test was reportedly high in women with pregnancy-related PPGP (information coefficient 0.82) [44].

#### Maximum Isometric Force

Maximum isometric muscle force measures will be obtained with a portable digital force gauge (SF-500, Akurasi, R.O.C). It will be periodically calibrated in accordance with the manufacturer’s information manual.

##### Maximum Isometric Hip Flexion Force

A digital force gauge will be attached to the metal bar and adjusted to be placed immediately above the ankle. The participant will be asked to raise their involved leg and compress the force gauge probe while their leg is still lying on the table [15]. The maximum isometric hip flexion force test correlates well with the ASLR test. The test will be repeated 3 times at a 20-second interval. The mean value will be recorded.

##### Maximum Isometric Hip External Rotation Force

The participant will undergo isometric muscle strength testing for external hip rotation using a digital force gauge and a stabilization strap. He/she will be asked to sit upright on the chair with his/her hip and knee positioned in approximately 90° flexion [45]. The stabilization strap will be placed immediately proximal to the ankle joint of the involved limb and fastened firmly around the chair’s leg. A force gauge will be secured between the medial side of the leg and the stabilization strap. The participant will be instructed to pull his/her leg inward with maximal effort until the force value is displayed on the force gauge. The test will be repeated 3 times at a 20-second interval. The mean value will be recorded [45]. The maximum isometric hip external rotation test was reported reliable in healthy subjects (SE 3.9, ICC 0.88) [45].

##### Maximum Isometric Trunk Rotation Force

The participant will be positioned on the chair in the sitting position with feet resting on the floor. Two nonelastic belts will pass diagonally over the chest and shoulder and finally are wrapped and secured around the back of the chair. Another belt will be fastened on the thighs to prevent extraneous pelvic movement. The force gauge will be fixed between the subclavicular area and the diagonal strap at one side. The participant will be asked to rotate his/her trunk toward the opposite side and exert isometric force to the force-probe, held in this position for 5 seconds. The test will be repeated thrice at a 20-second interval. The mean value will be recorded. The test will be performed on the opposite side using the same procedure [46]. A portable dynamometer provides suitable reliability and validity values to test different trunk muscles and populations [47]. Adequate reliability for the measurement of trunk rotator muscles was presented in subjects with stroke (ICC 0.64-0.99, SEM was considered low) [46] and older adults (ICC≥0.75) [48].

#### JPR of Hip Abduction

Hip proprioception will be measured using the active JPR while standing. The participant will stand with closed eyes on the uninvolved leg on a 10-cm-high wooden block. The involved leg will be allowed to freely move and abduct the hip joint. The participant will maintain his/her balance throughout the tests by touching a horizontal bar at hip joint height. During the first trial, the examiner will sit behind the participant and check the reference position and target angle. The hip abduction angle will be quantified using a large protractor attached in front of the participant on the wall. The protractor and hip joint center (greater trochanter) will be matched before starting the trial. The reference position is when the tested leg is placed parallel to the supporting limb in which the medial side of the tested leg is adjusted on the zero degrees of the protractor. The participant will randomly select the target angle within 10° to 40° for 4 trials. Three reflective markers will be attached to the apex of the iliac crest, greater trochanter, and lateral femur epicondyle [49]. The movement of the reflective markers will be recorded using a Canon camera (EOS-500D, DS126231) placed behind the participant at a distance of 2.5 m. The participant will be asked to actively move his/her leg from the reference position to the selected target angle at their self-selected velocity.

In the first trial, the examiner will instruct the participant on the “STOP” command to inform him/her of reaching the target angle. The participant will hold their leg at the target angle for approximately 4 seconds to memorize it. Then, the examiner will ask the participant to return his/her leg to a reference position of 0° by saying “Return” and holding the leg there for 3 seconds. Next, the participant will be asked to actively reproduce the previous target angle 3 times [49]. The camera’s tracking angles will be analyzed using Kinovea software (Version 0.9.2). Kinovea software is a valid and reliable tool to measure angle and distance accurately [50]. Intrasession reliability for angular error in hip abduction movement in healthy adults was reported between 0.39° and 0.96°, indicating a lower measurement error or more precise score for the hip joint proprioception test [49].

#### Demographic and Clinical Data

Demographic and clinical characteristics, including age, weight, height, BMI, duration of symptoms, and involved limb, will be prospectively collected and recorded on report forms.

### Data Analysis

All data will be saved with identification codes to maintain confidentiality. Every effort will be made to follow up on all subjects by the examiner. All enrolled participants, including those who do not complete treatment, will be included in the analysis on the basis of the intention-to-treat principle. Missing data will be replaced by the mean imputation technique, in which the mean of the observed values will be calculated and reported. Descriptive statistics will present demographic characteristics and outcome measures for each intervention group. Multivariate ANOVA will be used to test between- and within-group differences. Data will be controlled for outliers and checked for the normality and homogeneity assumptions. Post hoc (Bonferroni) tests will be conducted to report pair-wise comparisons. SPSS (version 17.0; SPSS, Inc) will be used for statistical analysis, and statistical significance will be set at a *P* value of <.05.

## Results

This study will start in summer 2022 and be complete by fall 2022.

## Discussion

### Expected Findings

It is generally accepted that rehabilitative modalities, including pelvic support, are suitable for PPGP [17]. Previous research has shown that lumbosacral orthosis effectively improved motor control and lumbopelvic function among patients with lower back pain [22]. The pathomechanics of lower back pain and PPGP are similar and include dysfunction of load transfer across the pelvis; hence, it has been hypothesized that wearing lumbar support could have beneficial effects for women with PPGP.

This study protocol will compare the impact of pelvic and lumbar supports on pain, disability, and hip repositioning in postpartum PPGP. The study results can be helpful for clinical decision-making. We anticipate that the study results will have significant implications for practitioners and specialists by increasing the scientific basis and contributing to the successful implementation of effective rehabilitation programs in clinical settings. Earlier studies have shown that both pelvic compression belts in pregnant women with PPGP [28] and lumbosacral orthosis in low back pain can significantly decrease pain [51] and improve proprioception [52], which can be explained by the biomechanical support provided [53] and proprioceptive impact of the orthosis [22]. At present, it is impossible to quantify which support is superior in reducing pain and improving proprioception and quality of life in women with PPGP. Suppose the lumbar support offers more favorable effects than the pelvic support in terms of pain reduction and improved sensorimotor outcomes. In that case, new orthotic interventions can be advised for women with postnatal PPGP.

This clinical study will evaluate repositioning in women with PPGP. For this repositioning, the JPR will be tested in a standing position. The upright position is a functionally relevant condition often occurring during daily activities. The hip abduction was selected since more precise scores for the proprioception test for the hip joint are assessed during this movement [49]. If pelvic and lumbar supports could affect the pain or load transfer at the SIJ, it could be reflected in the precision of JPR. The maximum force generation during trunk and external hip rotation is planned to quantify the possible effects of wearing the pelvic or lumbar support on the load transfer across the lumbopelvic area.

### Strengths and Limitations

The strength of this study will be the investigation of the effectiveness of newly designed lumbar support for the management of PPGP. There may be limitations to this study. The inclusion and exclusion criteria set in this study can limit the generalizability of the results. Another limitation could be that some participants may be excluded because of incomplete trial time or not being interested in wearing the support.

## Appendix

Multimedia Appendix 1 Peer-review reports from the Isfahan University of Medical Sciences - Office for Research and Technology (Isfahan, Iran).

## Abbreviations

ASLR: active straight leg raise
CONSORT-EHEALTH: Consolidated Standards of Reporting Trials of Electronic and Mobile Health Applications and onLine TeleHealth
ICC: intraclass correlation coefficient
IUMS: Isfahan University of Medical Sciences
JPR: joint position reproduction
ODI: Oswestry Disability Index
PPGP: posterior pelvic girdle pain
SIJ: sacroiliac joint
TIDieR: Template for Intervention Description and Replication
VAS: visual analogue scale

## Data Availability

The data sets will be included in the main manuscript and available from the corresponding author at reasonable request.
