# Supplementary material for: The Efficacy of Lumbar Support on Pain, Disability, and Motor Control in Women With Postpartum Pelvic Girdle Pain: Protocol for a Randomized Controlled Trial
Source: JMIR Res Protoc. 2022 Jul 20;11(7):e40553. doi: 10.2196/40553 (PMC9350821; doi:10.2196/40553)
Supplement: Multimedia Appendix 1 [file researchprotocols_v11i7e40553_fig.pdf]

Isfahan University of Medical Sciences
Vice-Chancellor for Research and Technology

Research Grant Peer review Form

**Funding Source**
Postgraduate studies and research program
**Amount**
253,500,000 Rials
**Electronic Submission Code**
3991082
**Principle investigator**
Ebrahim Sadeghi Demneh
**Co-investigators**
Fahimeh Sadat Jafarian, Mahmonir Jafari-Harandi, Gillian Yeowell
**Project title**
The efficacy of Lumbar support on Pain, Disability, and Motor Control in Women with Postpartum Pelvic Girdle Pain: Protocol for a Randomized Controlled Trial

Dear Ebrahim Sadeghi Demneh

Thank you for submitting your manuscript to the university's office for research and technology. We have received comments from the reviewers on your proposal. Reviewers have included some feedback on the different sections of the manuscript and hope you find these comments helpful.

Your research proposal should become acceptable for registration, pending suitable revisions and modifications of the proposal in light of the appended reviewer comments. Note that it may still be necessary to receive feedback from the reviewers prior to making a final decision.

When resubmitting your manuscript, please carefully consider all issues mentioned in the reviewers' comments, outline every change made the point by point, and provide suitable rebuttals for any comment not addressed.

To submit your revised manuscript go to https://researches.mui.ac.ir/index.phtml and log in as an academic member, where you will see a menu item called 'Proposal Needing Revision'.

Please resubmit your manuscript by Feb 22, 2022.

Reviewers' comments:

Introduction

- Consider clarifying the second aim related to "load transfer."
- Adding an explanation of how the lumbar support change on load transfer.
- The reference number "1" appears to be old. Please replace it with a new reference.

Method

- Providing reference(s) for test-retest reliability of isometric muscle force tests would be helpful.
- Which accurate diagnostic tests best differentiate patients with "low back pain" from those involved in "sacroiliac joint pain"? Suggest providing tests in detail.
- In diagnostic tests, it should be clarified that more than three tests must be done to verify sacroiliac joint involvement.
- Consider inserting the word "unilateral" in sacroiliac joint involvement.
- The method of measuring size, pelvic belt, and lumbar support should be explained.
- Please add some details on the timelines of lumbar support (posteriorly and anteriorly). More information on wearing the supports to obtaining maximum comfort would also be helpful.
- Please provide the accuracy of the instrument you considered for the "joint position reproduction of hip abduction" outcome.
- It appears that the only Oswestry disability index questionnaire is well enough to assess disability in involved participants. Suggest the functional activity could be eliminated.
- Please define the intensity and duration of the pain in the inclusion criteria.
- Consider adding limb length discrepancy in the exclusion criteria.
- Please explain which outcome could assess the load transfer.
- It would be best to add trunk movements like rotation to evaluate the efficacy of support. The author(s) mentioned only one direction (hip flexion).
- Consider adding a suitable reference for the visual analog scale.
- Please consider a minimum score in "perceived effort during the active straight leg raising test" for included participants.
- Providing reference(s) for test-retest reliability of instruments would be helpful.

Discussion

- It may be best to explain how to measure motor control assessment.
- Please add details about the efficacy of lumbar support on trunk muscles.
- Please explain why hip abduction movement in a standing position is considered for the selected outcome.
